# PAREsnip2: a tool for high-throughput prediction of small RNA targets from degradome sequencing data using configurable targeting rules

**DOI:** 10.1093/nar/gky609

**Published:** 2018-07-11

**Authors:** Joshua Thody, Leighton Folkes, Zahara Medina-Calzada, Ping Xu, Tamas Dalmay, Vincent Moulton

**Affiliations:** 1 School of Computing Sciences; 2 School of Biological Sciences, University of East Anglia, Norwich NR4 7TJ, UK

## Abstract

Small RNAs (sRNAs) are short, non-coding RNAs that play critical roles in many important biological pathways. They suppress the translation of messenger RNAs (mRNAs) by directing the RNA-induced silencing complex to their sequence-specific mRNA target(s). In plants, this typically results in mRNA cleavage and subsequent degradation of the mRNA. The resulting mRNA fragments, or degradome, provide evidence for these interactions, and thus degradome analysis has become an important tool for sRNA target prediction. Even so, with the continuing advances in sequencing technologies, not only are larger and more complex genomes being sequenced, but also degradome and associated datasets are growing both in number and read count. As a result, existing degradome analysis tools are unable to process the volume of data being produced without imposing huge resource and time requirements. Moreover, these tools use stringent, non-configurable targeting rules, which reduces their flexibility. Here, we present a new and user configurable software tool for degradome analysis, which employs a novel search algorithm and sequence encoding technique to reduce the search space during analysis. The tool significantly reduces the time and resources required to perform degradome analysis, in some cases providing more than two orders of magnitude speed-up over current methods.

## INTRODUCTION

Small RNAs (sRNAs) are short, non-coding RNAs that are vital components of gene regulation acting through endogenous RNA silencing pathways. They regulate many important and diverse biological pathways such as growth and development, disease resistance, and stress response (1,2). To do this, they suppress the translation of messenger RNAs (mRNAs) by directing the RNA-induced silencing complex (RISC) to its sequence-specific mRNA target(s). They can be classified into several classes such as microRNA (miRNA) and short interfering RNA (siRNA), differentiated by both biogenesis and mode of action (3). In plants, a high degree of complementarity between the sRNA and its mRNA target typically results in the endonucleolytic cleavage and subsequent degradation of the targeted mRNA (4).

An important step in understanding the biological function of a sRNA is to identify and validate its targets. Most computational tools for plant (and animal) sRNA target prediction use techniques that search for complementarity between a sRNA sequence and a potential target-sequence (5). These types of prediction use stringent, position based targeting rules that tend to report a high number of predictions and offer little flexibility. Whilst these results will almost certainly contain genuine targets, many of the predictions may be false positives (5). Therefore, the predicted targets must undergo further experimental validation through low-throughput techniques such as 5′ rapid amplification of cDNA ends (RACE) (6).

In the last few years, three high-throughput sequencing techniques (parallel analysis of RNA ends (PARE) (7), genome-wide mapping of uncapped and cleaved transcripts (GMUCT) (8) and degradome sequencing (9)) have become a high-throughput alternative for identifying sRNA mediated cleavage products on a genome-wide scale. They capture the uncapped 5′ ends of cleaved mRNA sequences giving a snapshot of the mRNA degradation profile, often termed the degradome. The cleaved mRNA fragments can then be aligned back to the reference transcript and used as evidence for sRNA mediated cleavage.

CleaveLand (10) was the first tool to use this approach for analysing degradome data. It has been used to successfully identify sRNA targets in a number of plant species (11–14) using a mismatch-based scoring scheme inferred from a set of experimentally validated miRNA-target interactions in *Arabidopsis thaliana* (15). Subsequently, Sequencing-based sRNA Target Prediction (SeqTar) (16) was developed in an attempt to loosen the stringent targeting rules implemented within CleaveLand. These rules, such as restrictions on the number of mismatches allowed within a sRNA–mRNA duplex and strictly not allowing a mismatch or G:U wobble pair at position 10 or 11, may result in CleaveLand discarding genuine interactions. SeqTar was shown to have higher accuracy than CleaveLand when using a less stringent set of targeting rules (16), but it is not publicly available to download or use.

Both CleaveLand and SeqTar suffer from the same restrictions due to their underlying algorithms. In particular, they are only able to perform an analysis on a small set of input sequences, such as known miRNAs, or a limited number of candidate sRNAs, without considerable time constraints. This led to the development of PAREsnip (17), which is an accelerated approach to degradome analysis that is able to process entire sRNA datasets within a feasible time frame on a typical desktop computer. Since its release, PAREsnip has been successfully used for genome-wide analysis on a number of different plant species (18–20). However, PAREsnip implements the same stringent targeting rules as CleaveLand and relies on complementarity at position 10 and 11 of the sRNA for its speed. Furthermore, based on our computational benchmarking, PAREsnip requires a considerable amount of computational resources when performing analysis on larger sequencing datasets.

Small RNA-PARE Target Analyzer (sPARTA) (21) is the most recent tool for degradome analysis. Unlike CleaveLand and PAREsnip, it does not assume a positive correlation between complementarity in the canonical seed region (2–13 nt from the 5′ end of the miRNA) and probability of actual cleavage. It offers the user two scoring schemes during the target prediction process: standard and seed free. The first is based on the analysis of experimentally validated targets and the complementarity rules based on the seed region (22). The second, which is also the default scoring system, allows for more flexibility within the seed region of the sRNA-target duplex and is based on genuine miRNA target interactions that differ from the canonical targeting rules (16,23). Whilst sPARTA offers more flexibility when searching for targets, based on our computational benchmarking, it suffers from the same time restrictions as CleaveLand and has high computational resource requirements.

Recent advances in high throughput sequencing technologies has resulted in larger, more complex genomes being sequenced such as *Pinus taeda* (24) *or Triticum aestivum* (25), both being many times larger than that of popular model organisms. Moreover, not only are larger genomes being sequenced, but degradome and sequencing datasets in general are growing ever larger in size and read count, with a typical sequencing experiment now containing millions of distinct reads in a single sample. In addition, the need for multiple samples and replicates is becoming the *de-facto* standard for biological experiments, further adding to this sequence-data deluge.

All of the tools for degradome analysis mentioned above are unable to process the volume of data currently being produced without imposing considerable time and resource constraints. In addition, the accuracy of these tools is primarily determined by the targeting rules that they apply and each tool uses a different set of fixed rules, which reduces their flexibility. Indeed, the rules currently implemented by the tools are inferred from the analysis of experimentally validated miRNA targets in *A. thaliana*. This was first performed on 94 validated miRNA-target duplexes by Allen *et al.* (15) and then, through a similar approach, on a larger set of 155 validated target duplexes by Fahlgren and Carrington (22). As our understanding of miRNA targeting improves, these rules may change, and so current tools risk becoming obsolete.

In this paper, we introduce a novel degradome analysis method and software tool, which we call PAREsnip2, that is scalable with current sequencing datasets. As we shall see, PAREsnip2 has greater predictive power than previous tools and also provides a vast reduction in computation time and resource requirement. Additionally, PAREsnip2 enables users to perform degradome analysis using configurable targeting rules. We shall illustrate the tool's use by analysing recently sequenced *A. thaliana* datasets. Although PAREsnip2 uses a different approach, we give it this name since it is freely available in the UEA sRNA Workbench (26) where its predecessor, PAREsnip (17), is also implemented.

## MATERIALS AND METHODS

**Figure 1.**
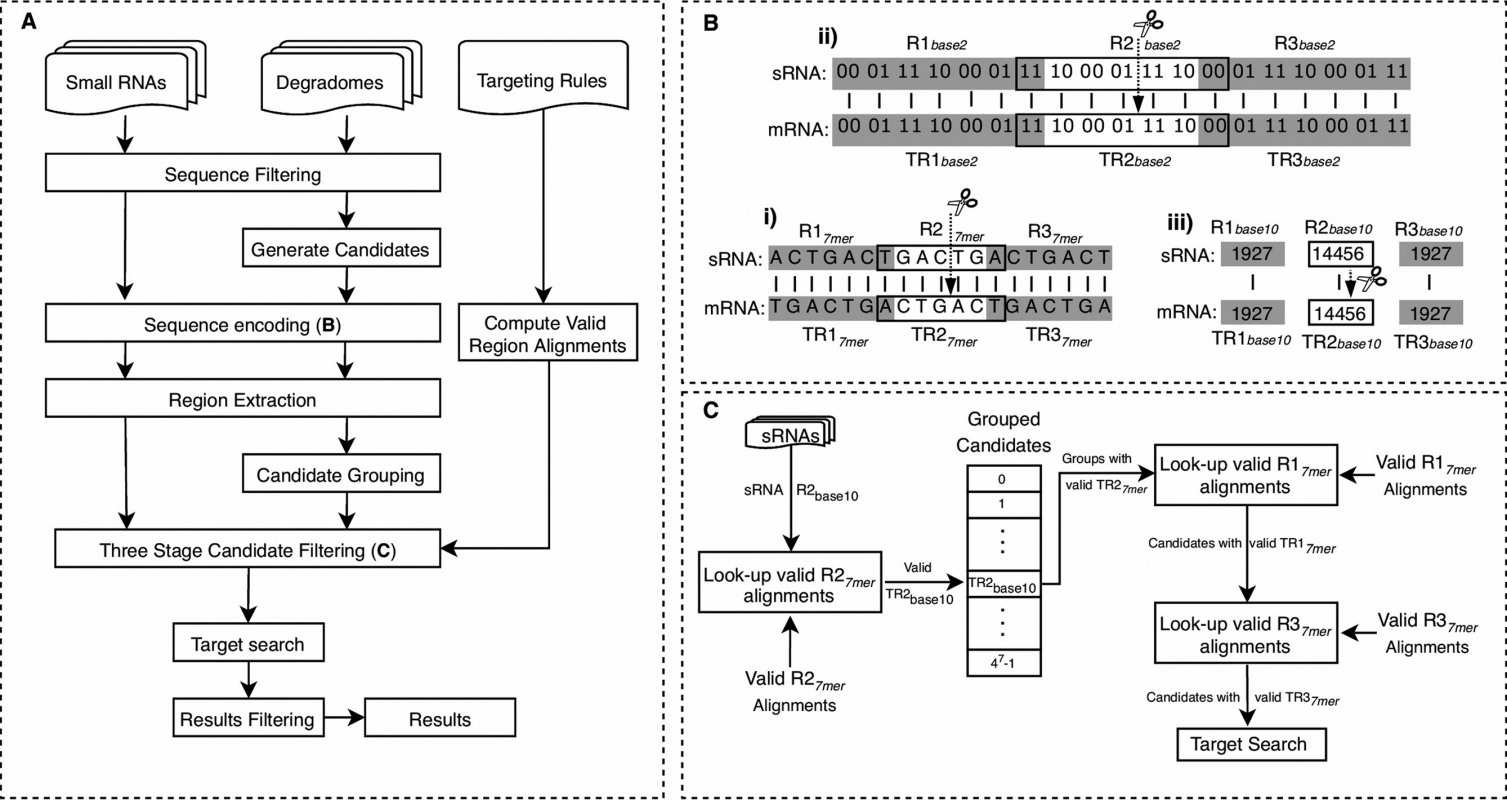
An overview of different stages of the PAREsnip2 algorithm. (**A**) Shows the inputs and processing steps performed to predict sRNA targets evidenced through degradome sequencing. (**B**) Shows the process of encoding sequence data into a number system. (**C**) Visual representation of the three-stage candidate filtering process. Regions are labelled R and target regions are labelled TR.

The PAREsnip2 algorithm is split into three main stages. The first stage is the input of the sequencing data and targeting rules, the second is the pre-processing steps (developed to improve the speed and efficiency of an analysis), and the third is the prediction of sRNA targets. A visual overview of the steps involved in performing an analysis on the input data is shown in Figure 1A. We now explain each stage of the algorithm in more detail.

### Data input

To perform an analysis using PAREsnip2 for a specific organism, the user must input the following data:

- a reference file (transcriptome) in either FASTA format or Generic Feature Format version 3 (GFF3) with corresponding genome;
- a genome file (optional unless using GFF3 as reference);
- one or more sRNA library replicates;
- one or more degradome library replicates

A reference file and at least one sRNA and degradome library are required to perform an analysis. If the user chooses to use a GFF3 file as a reference then a corresponding genome must also be provided. When extracting the gene sequences from the genome using a GFF3, the user has the option to include or exclude untranslated regions (UTRs).

The sRNA and degradome libraries must be in redundant FASTA format with the adapters trimmed.

### Sequence filtering

Several optional filtering techniques can be applied to the input data to remove low quality reads, sequencing errors or sample contamination. First, any sequence containing ambiguous bases are discarded, as they cannot be accurately aligned. Second, a low complexity sequence filter is applied based on the sequence single, di- or tri-nucleotide composition. This works by discarding any sequences that contain more than 75%, 37.5% and 25% of a single, di- or tri-nucleotide composition, respectively. Third, we provide the functionality to filter sequences using conservation over multiple samples where sequences will only be considered if they are present within each sample. Finally, when a genome is provided, sRNA sequences can be aligned to the genome using PatMan (27), with any sequences that do not align being discarded.

### Binary encoding of sequence input

A core component of the PAREsnip2 algorithm is the encoding of sequence data into a number system (Figure 1B). Given that a sequence is composed of four nucleotide bases (A, C, G, T/U), it is possible to represent each nucleotide using two bits of computer memory (Table 1), known as the base 2, or binary representation, of a nucleotide. We represent a whole sequence as a single decimal number by concatenating the binary representations of each nucleotide and converting the binary representation into decimal. We use this encoding technique to reduce the memory footprint of storing sequence data in memory and to speed up analysis. Furthermore, sRNA and mRNA sequences have an inverse encoding (Table 1), such that if a sRNA and mRNA sequence are represented by the same number then they will be perfectly complementary.

**Table 1.** The 2-bit binary encoding of nucleotides within sequence data

Nucleotide base	sRNA Encoding	mRNA Encoding
A	0 0	1 1
C	0 1	1 0
T/U	1 1	0 0
G	1 0	0 1

### Target candidate generation

To search for potential sRNA targets, we first generate a set of potential target-sequence candidates from the input data. The alignment of the degradome fragments to the reference gene sequences can inform us of potential sRNA cleavage events, with higher abundance fragments at a specific position more likely to be true cleavage signals. We developed a novel technique for exact match sequence alignment that uses the sequence encoding described above. First, degradome sequences are read from file, encoded as a number, and stored into a list. Once all the reads have been encoded and stored, the list is sorted into ascending order. Next, we split the reference sequences into subsequences using a sliding window and encode each of these into a decimal number. The size of the sliding window and the number of extracted subsequences are dependent on the accepted size range of the degradome reads. We then search the sorted list of encoded degradome fragments for the encoded reference subsequence using a binary search. If the number representing an encoded subsequence is found, an exact match has been identified at that position and is recorded. Once each reference sequence has been searched, the aligned degradome fragments are further processed to generate the set of target-sequence candidates. From the alignment position, we take 16nt towards both the 5′ and 3′ ends, resulting in a 32nt mRNA target-sequence candidate.

The newly generated target-sequence candidates are then sorted into one of five categories based on those previously defined in CleaveLand (V4) (10) with a minor modification. In our modification, we do not consider those fragments with an abundance of 1 during the average coverage calculation. This helps us to distinguish true lower abundance peaks from background degradation upon the transcript. An overview of the category system is provided below:

- Category-0 peaks are those that have greater than one read and are the maximum on the transcript when there is only one maximum;
- Category-1 peaks are those that have greater than one read and are the maximum on the transcript, but there is more than one maximum;
- Category-2 peaks are those that have greater than 1 read and are above the average fragment abundance on the transcript;
- Category-3 peaks are those that have greater than 1 read and are below or equal to the average fragment abundance on the transcript;
- Category-4 peaks are those that have just one read at that position on the transcript

### Region extraction and candidate grouping

Three regions of length 7nt (7*mer*) are extracted from both the input sRNA sequences and the generated target-sequence candidates. These are named region R1, R2 and R3 for the sRNA and target region TR1, TR2 and TR3 for the target-sequence. The position of the extracted target-sequence regions are based on a potential cleavage position i.e. where the sRNA would align if there were no gaps or bulges within the duplex (Figure 1Bi). The extracted region sequences are then encoded into their decimal number format and stored for later use. Finally, the generated target-sequence candidates are grouped together using the decimal representation of their TR2 sequence such that any candidates sharing the same 7*mer* at their TR2 will be grouped together.

### Predefined and user configurable targeting rules

Since the discovery of miRNAs and their regulatory role in plants, there has been much discussion on the rules that should be used when predicting plant miRNA targets (15,21–23,28–31). To the best of our knowledge, there are two generally accepted targeting rules for plant miRNAs. These rules are implemented within a position dependent scoring system based on the number of mismatches, G:U wobbles and target-bulged bases within the duplex. The first of these were inferred by Allen *et al.* in 2005 (15) and the second, through a similar approach with a larger set of validated targets, by Fahlgren and Carrington in 2010 (22). During a PAREsnip2 analysis, the user can choose between two sets of default targeting rules, either the Allen *et al.* (15) rules or the Fahlgren and Carrington (22) rules. The difference between them is that the Fahlgren and Carrington rules permit a mismatch or G:U wobble at position 10 or 11 of the sRNA. However, these rules are based on a small set of experimentally validated miRNA targets and as more miRNA targets are experimentally validated, our understanding of these targeting rules may change. To address this, we offer the ability to search for potential targets based on a user configurable rule set. The rules that can be configured by the user and used during the search for potential targets are shown in Table 2.

**Table 2.** Features within a sRNA–mRNA alignment which are used during the duplex alignment process and can be configured by the user

Configurable Search Parameters	
Maximum score	Maximum adjacent mismatches
Maximum G/U Wobble Pairs	Maximum Mismatches
Mismatch Score	G/U Wobble Score
Gap Score	Permissible Mismatch Positions
Core Region Start Position	Core Region End Position
Maximum Mismatches Core Region	Maximum Adjacent Mismatches Core Region
Allow Mismatch Position 10	Position 10 Mismatch Score
Allow Mismatch Position 11	Position 11 Mismatch Score
Core Region Multiplier	Non-permissible Mismatch Positions
Max Gaps Allowed	G/U Wobble Counts as Mismatch

### Computing valid region alignment matrices

As discussed previously, we can represent biological sequences using decimal numbers. *7mers* that are comprised of a four-letter alphabet (A, C, G and T/U), where each nucleotide is encoded using 2 bits of computer memory, are represented by a decimal number between 0 and 16383. For each of the three regions, we create a 16384 × 16384 matrix that represents all possible combinations of alignments between 7*mers*. Within these matrices, row numbers represent encoded sRNA 7*mers* and column numbers represent encoded mRNA 7*mers*. The matrices are then populated by attempting to align the decoded sRNA and mRNA 7*mers* using the user's chosen set of targeting rules. If a valid alignment is found within the matrix, we set that position to true otherwise it is set to false. This is repeated for every possible combination of alignments between 7*mers* for each of the three regions.

### Three-stage candidate filtering

We developed a three-stage candidate filtering technique to reduce the search space and therefore the computation time required to perform an analysis. When searching for degradome peaks potentially resultant of sRNA mediated endonucleolytic cleavage, we use the valid region alignment matrices to discard candidates that do not fit the chosen targeting rules (Figure 1C). In the first stage of this technique, we consider only those target-sequence candidates where their TR2 7*mer* can successfully align to the R2 7*mer* of the sRNA. This is done by looking at the encoded sRNA R2 7*mer* row in the R2 valid region alignment table and taking all target-sequence candidates grouped on the columns set to true on that row.

In the second and third stages, we discard any target-sequence candidates where their TR1 or TR3 regions do not successfully align to the R1 or R3 regions of the sRNA. This is performed by first looking at the cell *(R1_base10_, TR1_base10_)* in the R1 valid region alignment matrix to see if it is set to true and if so, we do the same for the R3 and TR3 region, discarding any candidates if the cell values are set to false.

### Target search and results filtering

Any target-sequence candidate that passes all stages of the three-stage candidate filtering process is aligned to the sRNA sequence using our duplex alignment algorithm employing the chosen targeting rules. When attempting to align a sRNA to a potential target-sequence candidate, the search process starts at the cleavage site and then traverses towards the 5′ end of the sRNA and at each position performs a nucleotide comparison between the two sequences. If the alignment towards the 5′ end is successful, it then performs the same process towards the 3′ end. If there is a mismatch, it will attempt to insert a gap and continue the alignment. If at any point one of the user's selected rules are broken then the alignment is discarded. This process will find all valid alignments based on the chosen targeting rules and the best possible alignment is selected. We first attempt to select the alignment that has the lowest alignment score and if there are multiple valid alignments with this score, the alignment with the fewest gaps is reported. If there are multiple alignments with the same number of gaps, the alignment with the fewest number of mismatches and G:U wobble pairs is reported.

Once a potential target has been identified, two optional filtering processes can be performed to improve the confidence level of each prediction. The first is the application of a minimum free energy (MFE) ratio filter and the second is a *P*-value filter. The MFE is calculated using RNAplex (32,33) which was shown to score favourably for sensitivity and precision when compared to other similar methods in a recent benchmarking of performance (34). The MFE ratio is calculated by dividing the predicted target duplex MFE by the MFE of a perfectly complementary target site. Any predicted target site that has a MFE ratio less than a given cut-off is discarded. The default cut-off ratio is 0.7, as suggested by Allen *et al.* (15), but can be configured by the user. The second optional filtering process uses the binomial distribution *P*-value system implemented within CleaveLand V4 (10) but with the modification that the probability is calculated on a transcript by transcript basis.

### Implementation and output

The algorithm has been implemented using the Java programming language and a user-friendly, cross-platform software package has been incorporated into the UEA sRNA Workbench (26). Analysis can be performed through the graphical user interface (GUI) or through the command-line interface (CLI) allowing PAREsnip2 to be used in other bioinformatics pipelines or workflows.

The results of PAREsnip2 are provided in comma-separated value (CSV) format, allowing them to be viewed in any CSV file viewer. They include information about the transcript peak such as cleavage position, abundance and weighted-abundance at the cleavage site, and the category of the peak on the transcript. A visual representation of the sRNA–mRNA duplex is displayed along with its alignment score. The sequence read abundance for small RNA and degradome data are provided in both raw and normalized values so that sequencing libraries can be compared.

### Degradome library construction

Three *A. thaliana* degradome replicates were constructed using wild type Columbia (Col-0) plants grown at 22°C with 16 h light and tissue was harvested when plants were at growth stage 5, as defined by Boyes *et al.* (35). For each replica, RNA was isolated from a pool of all leaves taken from nine plants with TRI reagent following manufacturer's instructions. This RNA was then used to construct degradome libraries following Zhai *et al.* protocol (36), with the only difference being that SuperScript II reverse transcriptase was used instead of Superscript III.

### Sequence datasets

The transcriptome used in all of our analyses on *A. thaliana* was the TAIR10 cDNA 20110103 representative gene model updated (37).

The computational performance benchmarking was carried out using a publicly available *A. thaliana* mature leaf degradome dataset (38) obtained from GEO (39) (GSM1330562) which we shall call dataset D1. Additionally, we simulated 9 sRNA datasets of increasing size to use as input data. These sRNAs were generated by first aligning the D1 reads to the reference and then extracting 19–24 nt sequences centred on cleavage positions. Transcripts, cleavage positions and sRNA sequence lengths were selected at random.

The prediction performance benchmarking was performed using the three *A. thaliana* degradome replicates, which we described above, and *A. thaliana* mature miRNA sequences obtained from miRBase (v21) (40).

To perform genome-wide degradome analyses on *A. thaliana*, we obtained the corresponding sRNA libraries, which were previously published by our lab (41) (GSE90771), for each of the *A. thaliana* degradome replicates. Collectively we shall call this dataset D2 and refer to each individual degradome replicate as D2A, D2B and D2C hereafter. Additionally, we performed a genome-wide analysis on Triticum aestivum using publicly available sRNA (GSE36867) and degradome (GSE37134) datasets (42) and the Triticum aestivum transcriptome (cDNA) obtained from Ensembl Genomes (release 38) (43).

## RESULTS

### Sequencing data

We processed the raw data using tools provided within the UEA sRNA Workbench (26). The adapter trimming tool was used to trim the adaptor sequences in each of the three degradome replicates. Next, using the Filter tool, we discarded sequences that contained any ambiguous bases and aligned the remaining sequences to the genome (TAIR10) with no mismatches allowed. When mapping to the genome, 81%, 82% and 82% of trimmed reads successfully aligned in replicates D2A, D2B and D2C, respectively. Table 3 gives a summary of the statistics for the three replicates and Supplementary Figures S1–S3 show the read length distributions.

**Table 3.** Summary statistics from the sequencing of three *Arabidopsis thaliana* degradome replicates (NR = non-redundant)

Replicate	Untrimmed Reads	Untrimmed Reads (NR)	Trimmed Reads (NR)	Invalid Sequences Filtered (NR)	Genome Matched Reads	Genome Matched Reads (NR)
D2A	45 581 525	15 267 190	11 114 679	21 004	41 144 941	9 009 977
D2B	34 915 085	13 385 729	10 103 828	17 049	31 426 832	8 316 470
D2C	26 067 832	10 199 905	7 715 372	12 140	23 303 530	6 337 667

### Computational performance benchmarking

To measure the computational performance of the PAREsnip2 algorithm i.e. the time and memory required to perform an analysis, we carried out computational benchmarking and compared our results to those of other publicly available methods. This benchmarking was performed on a desktop computer running Ubuntu 16.04 equipped with a 3.40GHz Intel Core i7-6800K six core CPU and 128GB RAM. Each tool was run using the authors default suggested parameters and for the fairest comparison, we included all filtering and pre-processing options available in PAREsnip2. Additionally, we set the number of threads to be used by the tools during the analyses to 12, except for CleaveLand as it was not an option.

For this benchmarking, we used the D1 dataset, the simulated sets of sRNA sequences and the TAIR10 cDNA transcriptome. Whilst the tools were performing the analysis on the simulated data, we monitored their peak memory usage and recorded the time they took to complete the analysis. The results of these analyses for both time and peak memory usage is shown in Table 4. Additionally, if the tool did not complete the analysis within 10 days, we recorded it as did not finish (DNF).

**Table 4.** Benchmarking results for both time and memory usage in Gigabytes (GB) from running each tool using the generated small RNA datasets. If the entry is DNF it means that the tool did not complete the analysis within the 10 day cut-off. A ‘-’ means that we did not attempt to run the tool

# Seqs	CleaveLand4	GB	PAREsnip	GB	sPARTA	GB	PAREsnip2	GB
1	19m 23s	1	9m 30s	58	12m 48s	25	5m 38s	5
10	27m 32s	1	9m 50s	58	12m 53s	25	5m 36s	5
100	1h 52m	1	12m 35s	58	13m 55s	25	5m 44s	5
1,000	15h 8m	1	44m 51s	58	1h 11m	26	6m 15s	6
10,000	6d 6h 48m	8	6h 25m	64	4d 6h 59m	37	6m 32s	6
100,000	DNF	-	2d 15h 16m	66	DNF	-	15m 1s	6
250,000	-	-	6d 10h 49m	68	-	-	29m 6s	7
500,000	-	-	DNF	-	-	-	53m 11s	8
1,000,000	-	-	-	-	-	-	1h 44m	8

The results show that the newly developed PAREsnip2 algorithm substantially outperforms all the currently available tools on the simulated datasets. The largest dataset for which any of the existing tools could process in under 10 days contained 250 000 sequences. When performing analysis on this dataset, PAREsnip2 showed over two orders of magnitude (∼300×) improvement in computation time. Additionally, the results suggest that the computation time of PAREsnip2 grows linearly with the number of input sequences, taking just 1 h and 44 min to process the largest of the simulated datasets (1 000 000 sRNAs).

### Prediction performance benchmarking

To evaluate the prediction performance of each tool we collected a set of experimentally validated *A. thaliana* interactions by combining those previously published in the literature (17,44,45) and those contained within miRTarBase (46) with any duplicates being removed. In total, we collected 616 validated interactions comprising 135 miRNAs. Out of these 135 miRNAs, 90 of them had unique sequences and were involved in 387 distinct miRNA–mRNA interactions. See Supplementary Table S1 for the complete list of curated validated targets.

Any of the validated interactions with a category-4 signal at the cleavage position on the transcript within the D2 degradome datasets were excluded from the benchmarking. These signals were excluded because it is difficult to distinguish between true miRNA cleavage products and random degradation with such low abundance. To identify the cleavage positions, we obtained the miRNA sequence from miRBase and the transcript sequence for each of the validated miRNA targets and performed the alignment between them using loose targeting rules (maximum seven mismatches). In the case that multiple alignments were found between the miRNA and its target, we retained the alignment(s) with the best alignment score and minimum free energy ratio. The position on the transcript opposite position 10 of the miRNA was recorded as the miRNA cleavage site. The category of the signal on the transcript was determined by aligning the D2 degradome datasets to the transcript and recording the abundance at the cleavage position. Out of a possible 387, we included 243, 239 and 224 validated interactions comprising 61, 60 and 58 miRNA sequences for datasets D2A, D2B and D2C, respectively.

We performed an analysis with each tool using the miRNA sequences contained within the validated set of miRNA–mRNA interactions, the *A. thaliana* transcriptome, and the three D2 degradome datasets described previously. Each tool was run using the default parameters recommended by the authors but with category-4 interactions discarded as they were not considered previously. When benchmarking PAREsnip2, we performed the analysis using both sets of default targeting rules and the MFE filter with cut-off score of 0.7. The results produced by each tool when analyzing the three datasets were then compared against the set of validated targets and are shown in Table 5. The results show that both sets of default targeting rules implemented within PAREsnip2 captured more of the experimentally validated interactions than the currently available tools. The differences between the results produced by the tools are likely due to variations in the implemented targeting rules and the filtering techniques applied. Additionally, the lower number of interactions reported by CleaveLand may be due to the way it handles degradome reads that map to multiple transcripts. If a degradome read aligns to more than one transcript, only one is randomly selected and reported by CleaveLand.

**Table 5.** The results from the accuracy performance benchmarking of each tool over the three biological replicates. V = validated targets, NV = non-validated and %PV = percentage of possible validated targets that could be found

	Replicate D2A			Replicate D2B			Replicate D2C		
Tool Name	V	NV	%PV	V	NV	%PV	V	NV	%PV
sPARTA	171	120	70%	169	121	70%	162	127	72%
PAREsnip	177	48	73%	179	50	75%	167	57	75%
CleaveLand4	88	20	36%	95	26	40%	87	25	39%
PAREsnip2 Allen *et al.*	193	41	79%	191	39	80%	181	33	80%
PAREsnip2 Fahlgren & Carrington	219	48	90%	219	43	91%	205	37	91%

### Evaluation of the optional filtering methods

**Figure 2.**
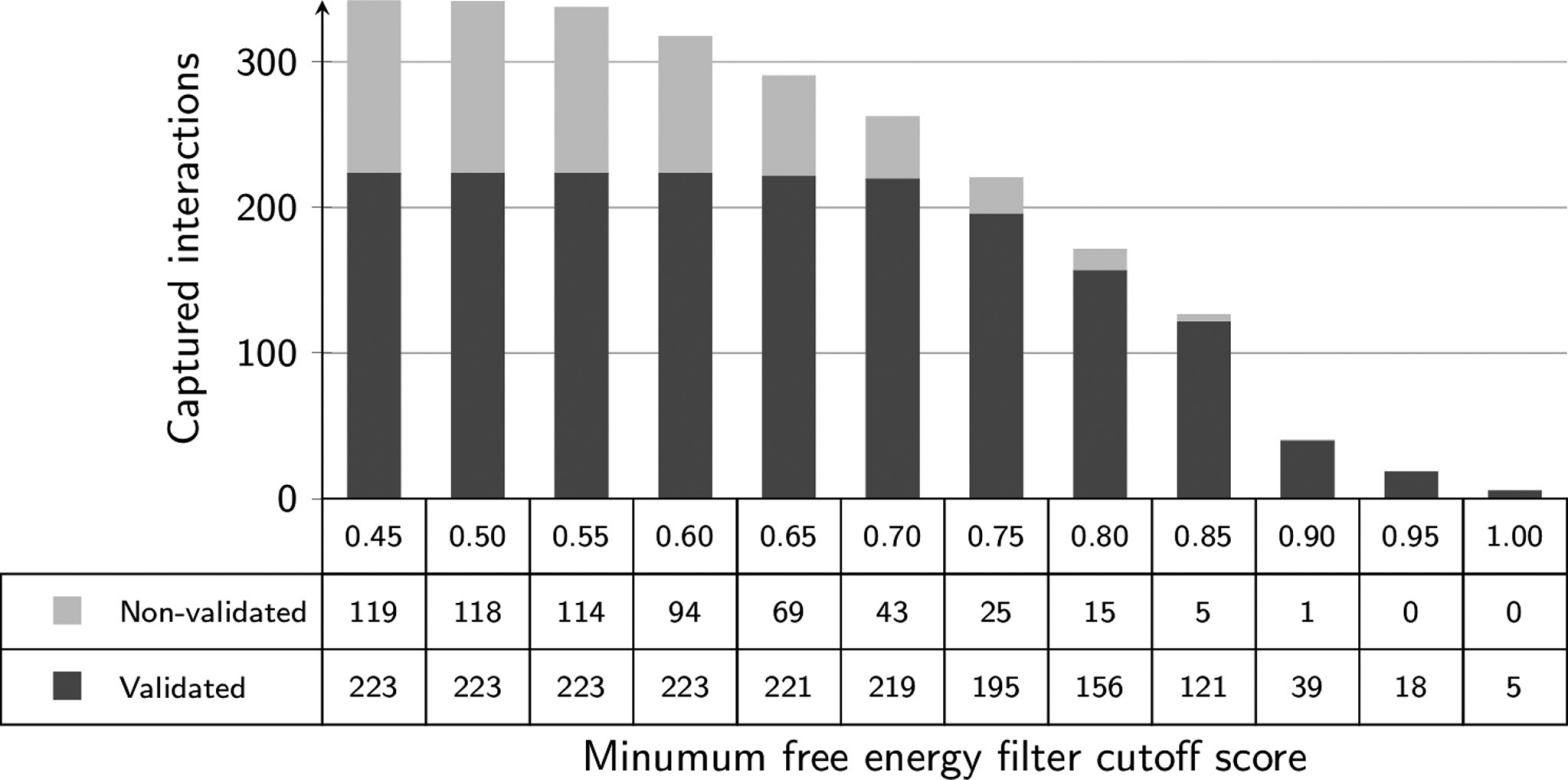
The number of interactions reported when using MFE as a filter. As the MFE filter ratio increases, there is a reduction in the number of captured sRNA–mRNA interactions. A cut-off score of 0.70 captures 98% of the possible validated interactions.

**Figure 3.**
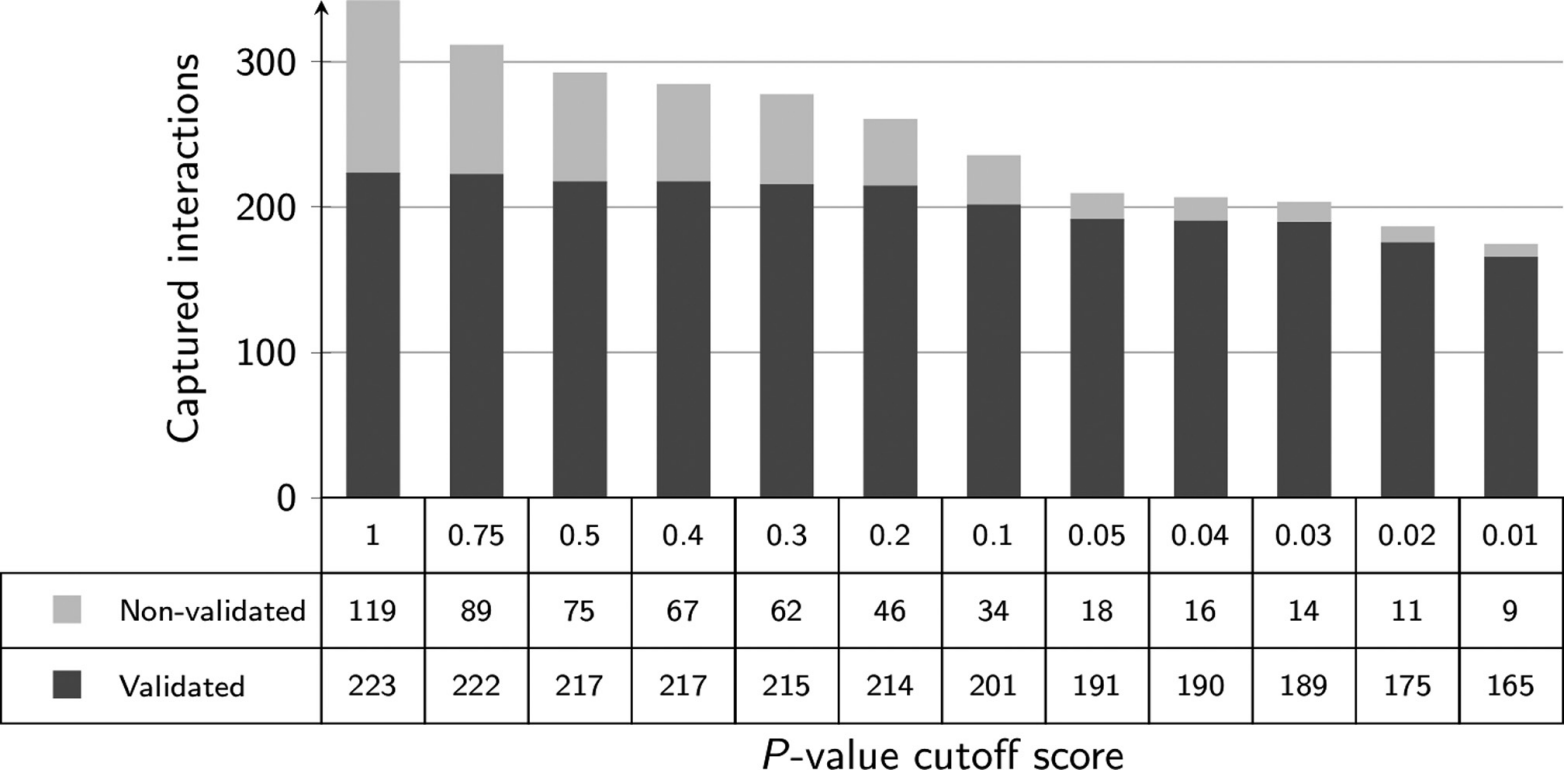
The number of interactions reported when using *P*-value as a filter. As the cut-off decreases, there is a reduction in the number of captured sRNA–mRNA interactions. The default cut-off score of 0.05 captures 85.6% of the possible validated interactions.

To evaluate the success of the filtering techniques implemented within PAREsnip2, we repeated the prediction performance benchmarking on the D2B degradome dataset using the 60 miRNA sequences, the default Fahlgren and Carrington targeting rules, and increasing filtering cut-off values. The results of the MFE analysis are shown in Figure 2 and the results of the *P*-value analysis are shown in Figure 3.

When evaluating the MFE filter, we start with a cut-off score of 0.45, as this captures all possible interactions, and with increments of 0.05 thereafter, we record the number of validated and non-validated targets being captured. Using the initial value, we captured a total of 342 miRNA–mRNA interactions from 60 miRNAs with 223 being part of the validated set and 119 were non-validated. At the other end of the scale, by using a filter cut-off value of 1 we captured just 5 interactions, all of which are part of the validated set. The default value of the MFE ratio filter (0.70) for PAREsnip2 captures a total of 262 interactions and of these the filtering process kept 219 (98%) from the possible 223 validated interactions.

Similarly, when evaluating the success of the *P*-value filter, we started with a cut-off score of 1, as this captures all possible interactions, and then repeated the analysis each time lowering the cut-off score and recorded the number of validated and non-validated targets being captured. A total of 342 interactions, with 223 validated and 119 non-validated, were captured using a cut-off score of 1 and a total of 174 interactions, with 165 validated and 9 non-validated, were captured using a score of 0.01. The default value for the *P*-value filter implemented within PAREsnip2 (0.05) captures a total of 209 interactions. Of these, the filtering process kept 191 from the possible 223 (85.6%) validated interactions.

### Genome-wide analysis of degradome datasets

To illustrate the use of PAREsnip2, we carried out a genome-wide scale degradome analysis of dataset D2 using the sRNA–mRNA target interaction rules as described by Allen *et al.* (15). For this analysis, we used the default stringent parameters, which discards category-4 signals and permits a minimum sRNA abundance of 5 reads. Additionally, the built-in conservation filter was used to increase confidence in the reported interactions. In total, PAREsnip2 captured 2008 sRNA–mRNA interactions (Supplementary Table S2), which comprised 960 category-0, 79 category-1, 511 category-2 and 458 category-3 interactions. To consider how the Allen *et al.* rules fared in capturing known interactions that have previously been validated, we compared the results with the set of curated validated targets (Supplementary Table S1). We found that 178 of the validated targets were conserved within the three replicates of the dataset (degradome signal and miRNA sequence), and of these the Allen *et al.* targeting rules captured 132 (74%), which were predominantly category 0 interactions. Interestingly, 46 of the validated interactions within the sequencing data were missed. This could have been due to the stringency of the parameters that were used, or that fact that the Allen *et al.* rules were based on a small set of experimentally validated interactions and are somewhat outdated in their representation of the requirements of miRNA mediated cleavage activity. Therefore, to test this we repeated the analysis on the same dataset but using the more recent Fahlgren and Carrington targeting rules, inferred in 2010, which allow mismatch and G:U wobble pairs at positions 10 and 11. This analysis identified 1072 category-0, 91 category 1, 611 category 2 and 529 category 3, making a total of 2303 interactions of which 151 (85%) of the possible validated interactions were captured (Supplementary Table S3). This shows a 11% improvement in identifying the known validated interactions over the Allen *et al.* targeting rules, which otherwise would have been missed. Performing this analysis using the Allen *et al.* rules took just 11 minutes and 32 seconds and the Fahlgren and Carrington targeting rules completed the analysis in 26 minutes and 48 seconds.

The timings for degradome analysis in *A. thaliana* led us to investigate the performance of PAREsnip2 on more complex species and larger genomes. The *Triticum aestivum* genome is much larger than *A. thaliana*, containing more than 155 000 transcript sequences within the genome annotation. We carried out a genome-wide analysis of the *T. aestivum* dataset (GSE36867), which comprised a degradome of 4 306 082 non-redundant (NR) sequences and a corresponding sRNAome of 14 133 641 NR sequences. The default stringent parameters identified 25 063 interactions (Supplementary Table S4), which comprised 12 120 category-0, 1026 category-1, 5576 category-2 and 6341 category-3 interactions and completed in just 31 minutes and 29 s. To investigate how using less stringent parameters would impact on the runtime performance of the tool, we repeated the analysis using the default flexible parameters. The tool identified 389,238 interactions (Supplementary Table S5), which comprised 83 409 category-0, 13 943 category-1, 79 935 category-2, 95 783 category-3 and 116 168 category-4 interactions with a runtime of 19 h and 39 min.

## DISCUSSION

In the age of genomics, the cost of sequencing has become cheaper and more accessible than ever before (47). This had led to many more genomes being sequenced, some of which are much larger and significantly more complex than popular model organisms. Many genomes are used in large scale studies from human health (48) to food production (49). Additionally, with the increasing number of reads being produced from sequencing experiments, the development of scalable and efficient algorithms for computational analysis of sequence data are becoming more and more important. We have developed a novel tool which is scalable with the increasing size and complexity of new genome releases and can perform a large scale degradome analysis using minimal computation resources. As an illustration, we ran our tool on wheat (*T. aestivum*), which in terms of base pairs is two orders of magnitude larger than *A. thaliana*. Using the default flexible parameters on the publicly available dataset described previously, the analysis took just 19 h and 39 min with a peak memory usage of 16GB and identified 389 238 targets by 169 636 sRNA sequences. In comparison, we terminated the execution of PAREsnip after 25 days on the same dataset, after which time it only reported 18% completion with a peak memory requirement of 175GB, far exceeding the resources you would expect to find in a typical desktop machine. Moreover, these results suggest that PAREsnip2 is the only tool capable of performing degradome analysis over multiple biological replicates within a reasonable time scale.

The miRNA targeting rules implemented within the currently available tools for degradome assisted target prediction are based on the analysis of experimentally validated miRNA targets in *A. thaliana*. These rules have been successfully applied to multiple other species during degradome analyses and sRNA target prediction with some predicted targets being further experimentally validated. However, probably in part due to the current lack of experimental evidence and to the best of our knowledge, no studies on miRNA targeting rules comparable to those performed on *A. thaliana* have been applied to other plant species. This may have resulted in overfitting our current understanding and implementation of these rules on *A. thaliana*. By providing the functionality to search for sRNA targets using configurable rules, users will be able to search for non-canonical targets that the existing rules would otherwise miss (16,21,23) and enable the potential to use a species specific set of rules if proven to be the case.

In its current form, PAREsnip2 is most suitable for the analysis of plant degradome datasets, as the primary mechanism for RNA silencing in plants is mRNA cleavage, whereas in animals the primary mechanism is translational repression. However, if the degradome data is available, PAREsnip2 could, in principle, be used for analysing sRNA mediated cleavage products in animals.

As is the case with many rule based systems, there exist a number of experimentally validated miRNA targets that do not fit the canonical set of targeting rules (16,21,23). By adjusting the parameters so that these targets are found, PAREsnip2 may run the risk of increasing the rate at which false positives are reported. One potential solution to this would be to perform an analysis using a less stringent set of targeting rules alongside the built-in conservation filter. For example, if a high confidence, i.e. high abundance and low category peak, miRNA-target is reported across multiple biological replicates then further investigation, such as other experimental validation techniques, could be used to confidently determine if the reported interaction is real.

The PAREsnip2 algorithm has been implemented into a user-friendly and cross-platform (Windows, Linux and MacOS) application that enables users to analyse their data without the need for dedicated bioinformatics support or specialized computer hardware. Additionally, the tool can be run using the command line for users who wish to incorporate PAREsnip2 into more complex computational pipelines. Enabling the use of specialist bioinformatics software without the need for any computational expertise will hopefully lead to new discoveries within RNA silencing pathways in all manner of experimental contexts.

## DATA AVAILABILITY

PAREsnip2 is available as part of the UEA sRNA Workbench and can be downloaded from http://srna-workbench.cmp.uea.ac.uk/. Additionally, the source code has been released on GitHub and is accessible at https://github.com/sRNAworkbenchuea/UEA_sRNA_Workbench/. The three degradome replicates have been submitted to GEO and can be accessed with accession code GSE113958.

## Supplementary Material

Supplementary Data
