# Supplementary material for: PAREsnip2: a tool for high-throughput prediction of small RNA targets from degradome sequencing data using configurable targeting rules
Source: Nucleic Acids Res. 2018 Jul 11;46(17):8730–9. doi: 10.1093/nar/gky609 (PMC6158750; doi:10.1093/nar/gky609)
Supplement: Supplementary Data [file gky609_supplemental_files.zip › user_manual.pdf]

Genome-wide analysis of degradome data using PAREsnip2

07/06/2018

User Guide

A tool for high-throughput prediction of small RNA targets from degradome sequencing data using configurable targeting rules

Joshua Thody, Leighton Folkes, Zahara Medina Calzada, Ping Xu, Tamas Dalmay and Vincent Moulton.

Contents

Introduction 2
System requirements 3
Input data, parameters and results 4
  Input data 4
  Parameters 4
    Analysis configuration 5
    Targeting rules 6
    Default parameters 7
Launching PAREsnip2 from graphical user interface (GUI) 9
  Loading input data 10
  Configuring analysis parameters 11
  Selecting targeting rules 12
Launching PAREsnip2 from the command-line (CMD) 13
PAREsnip2 results output 14

Introduction

Recent advancements in high-throughput sequencing technologies has resulted in larger, more complex genomes being sequenced. This has given us the potential to study species with much larger and more complex genomes than many popular model organisms. Moreover, sequencing datasets are growing ever larger in size and read count, with a typical sequencing experiment now containing millions of distinct reads. An important step in understanding the biological function of a small RNA (sRNA) is to identify and validate its targets. In plants, several classes of sRNAs have been shown to bind with near-perfect complementarity to their messenger RNA (mRNA) targets, generally leading to cleavage of the mRNA. High-throughput sequencing techniques have been developed to identify sRNA mediated cleavage products on a genome-wide scale. These techniques are generally referred to as degradome sequencing. Examples of these are parallel analysis of RNA ends (PARE) and genome-wide mapping of uncapped and cleaved transcripts (GMUCT). They capture the uncapped 5' ends of cleaved mRNA sequences giving a snapshot of the mRNA degradation profile, often termed the degradome. The cleaved mRNA fragments can then be aligned back to the reference transcript and used as evidence for sRNA mediated cleavage. PAREsnip2 takes one or more sRNA libraries, one or more degradome libraries and an mRNA dataset (or transcriptome) as input, and outputs the potential sRNA target duplexes evidenced through the degradome data using a configurable set of targeting rules.

System requirements

The UEA sRNA Workbench has been tested on various platforms including:

- Mac OSX (Version 10.5 Leopard; 10.6 Snow Leopard; 10.7 Lion, 10.8 Mountain Lion, 10.9 Mavericks, 10.10 Yosemite, 10.13 High Sierra)
- Linux (Ubuntu Version 16.04)
- Windows 7 and 10

Currently the software is built and tested on the official Oracle builds of Java only. However, most of the software should behave in the same way under open builds but we cannot guarantee this.

Required:

Java 8 (At the time of writing Java 9 is not yet supported)

Recommended:

Intel i5 quad core (or similar)

16GB RAM

Input data, parameters and results

This section discusses the types of data that can be used to perform degradome analysis with PAREsnip2. Additionally, we describe each parameter in detail.

Input data

To perform analysis using PAREsnip2 for a specific organism, the user must input the following data:

- a reference sequence (either a transcriptome or gff3 file with corresponding genome)
- a genome file (optional unless using gff3 as reference)
- one or more sRNA library replicates
- one or more degradome library replicates

A reference sequence and at least one sRNA and degradome library is required to perform the analysis. If the user chooses to use a gff3 file as a reference then a corresponding genome must also be included. When extracting the gene sequences from the gff3 and corresponding genome, the user has the option to include or exclude the UTRs.

The sRNA and degradome libraries must be in redundant FASTA format with the adapters trimmed. FASTQ to FASTA and adapter removal tools are provided within the UEA sRNA Workbench.

All of the sequence data must be given in non-redundant FASTA format. Additionally, sequences containing any ambiguous bases will be discarded as they cannot be accurately aligned.

Parameters

The parameters for PAREsnip2 are split into two categories, analysis parameters and targeting rules, and are set by the user before performing analysis.

Analysis configuration

Parameter	Values	Description
use_transcriptome	true/false	Flag to use transcriptome as reference
use_genome_gff	true/false	Flag to use genome and GFF3 to extract gene sequences
include_UTR	true/false	Flag to include UTR when exacting gene sequences
align_sRNAs	true/false	Flag to align the sRNAs to the genome
use_conservation_filter	true/false	Flag to perform conservation across multiple replicates
use_mfe_filter	true/false	Flag to use minimum free energy (MFE) ratio filtering
mfe_filter_cutoff	Decimal 0…1	Minimum MFE ratio for target to be reported
use_p_value	true/false	Flag to use p-value filtering
p_value_cutoff	Decimal 0…1	Maximum p-value score for target to be reported
filter_low_complexity_seqs	true/false	Flag to filter low complexity sequences
category_0	true/false	Flag to include category 0 peaks
category_1	true/false	Flag to include category 1 peaks
category_2	true/false	Flag to include category 2 peaks
category_3	true/false	Flag to include category 3 peaks
category_4	true/false	Flag to include category 4 peaks
number_of_threads	Number >= 1	Number of threads used during analysis. We recommend using the cores available x 2
min_sRNA_abundance	Number >= 1	Minimum abundance of sequence to be considered
min_sRNA_length	Number < 25	Minimum length of sequence to be considered
max_sRNA_length	Number < 25	Maximum length of sequence to be considered
min_fragment_length	Number >= 1	Minimum length of fragment to be considered
max_fragment_length	Number >= 1	Minimum length of fragment to be considered

Targeting rules

Parameter	Values	Description
allow_mismatch_position_10	true/false	Flag to allow a mismatch opposite position 10 of the sRNA
mismatch_position_10_penalty	Decimal 0…N	Penalty score for mismatch at position 10
allow_mismatch_position_11	true/false	Flag to allow a mismatch opposite position 11 of the sRNA
mismatch_position_11_penalty	Decimal 0…N	Penalty score for mismatch at position 11
gaps_count_as_mismatch	true/false	Flag to set gaps to count as mismatches
gu_count_as_mismatch	true/false	Flag to set G:U wobble pairs as mismatches
core_region_start	Number	Start position for the sRNA sequence core region
core_region_end	Number	End position for the sRNA sequence core region
core_region_multiplier	Decimal 0…N	Multiplier for mismatches, gaps and G:U pairs in the core region
max_adjacent_mismatches_core_region	Number	Max adjacent mismatches in the core region
max_mismatches_core_region	Number	Max mismatches in the core region
mismatch_score	Decimal 0…N	Score for a mismatch
gap_score	Decimal 0…N	Score for a gap
gu_score	Decimal 0…N	Score for a G:U wobble pair
max_score	Decimal 0…N	Maximum score for a report target duplex
max_mismatches	Number	Maximum mismatches in a reported target duplex
max_gu_pairs	Number	Maximum G:U pairs in a reported target duplex
max_gaps	Number	Maximum gaps in a reported target duplex
max_adjacent_mismatches	Number	Maximum adjacent mismatches in a reported target duplex
permissible_mismatches	1,2,3,...etc	Mismatch positions that are not penalised in the reported duplex
non-permissible_mismsatches	1,2,3,...etc	Mismatches that are **not** allowed in the duplex

Default parameters

Here are the default parameters in PAREsnip2. We offer two sets of default targeting rules based on the analysis of previously validated miRNA targets.

Default analysis configuration

Parameter	Stringent	Loose
use_transcriptome	true	true
use_genome_gff	false	false
include_UTR	false	false
align_sRNAs	false	false
use_conservation_filter	false	false
use_mfe_filter	true	true
mfe_filter_cutoff	0.7	0.7
use_p_value	true	true
p_value_cutoff	0.05	0.05
filter_low_complexity_seqs	true	true
category_0	true	true
category_1	true	true
category_2	true	true
category_3	true	true
category_4	false	true
number_of_threads	N/A	N/A
min_sRNA_abundance	5	1
min_sRNA_length	19	19
max_sRNA_length	24	24
min_fragment_length	20	20
max_fragment_length	21	21

Default targeting rules

Parameter	Allen et al.	Fahlgren & Carrington
allow_mismatch_position_10	false	true
mismatch_position_10_penalty	N/A	1
allow_mismatch_position_11	false	true
mismatch_position_11_penalty	N/A	1
gaps_count_as_mismatch	true	true
gu_count_as_mismatch	false	false
core_region_start	2	2
core_region_end	13	13
core_region_multiplier	2	2
max_adjacent_mismatches_core_region	1	1
max_mismatches_core_region	2	2
mismatch_score	1.0	1.0
gap_score	1.0	1.0
gu_score	0.5	0.5
max_score	4.0	4.0
max_mismatches	4	4
max_gu_pairs	4	4
max_gaps	1	1
max_adjacent_mismatches	2	2
permissible_mismatches	N/A	N/A
non-permissible_mismsatches	N/A	N/A

Launching PAREsnip2 from graphical user interface (GUI)

PAREsnip2 is a tool within the UEA sRNA Workbench. In order to run the sRNA Workbench in GUI mode, simply download the latest version from the UEA sRNA Workbench website and extract all the files from your downloaded zip archive to a new directory and then launch the Workbench.jar.

Next, click Open/Close menu -> Pre-configured Workflows -> Create PAREsnip2 Workflow

Note: If you would prefer to use the command line, please see section Launching PAREsnip2 from the command-line.

You will then be provided with the following screen

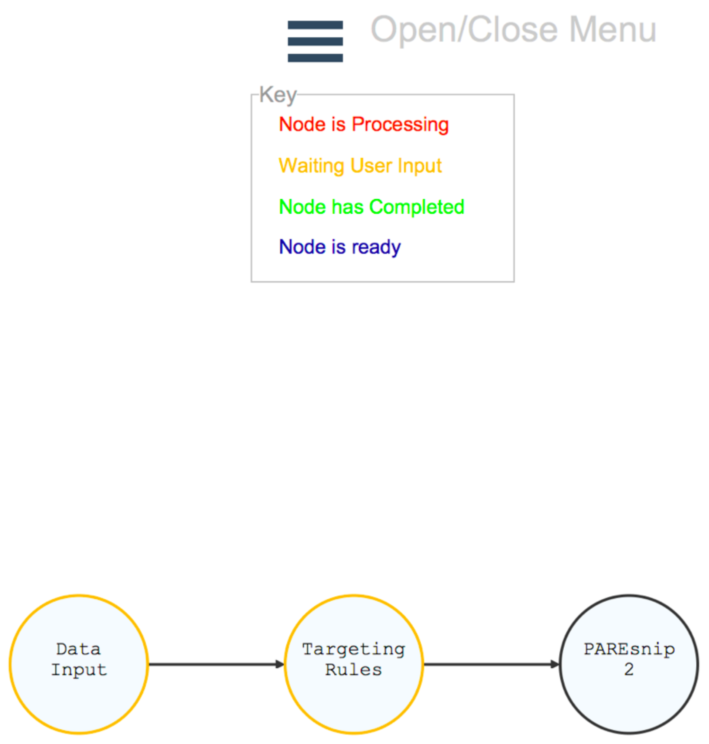

Loading input data

Double click on the 'Data Input' node and you will be provided with the following window

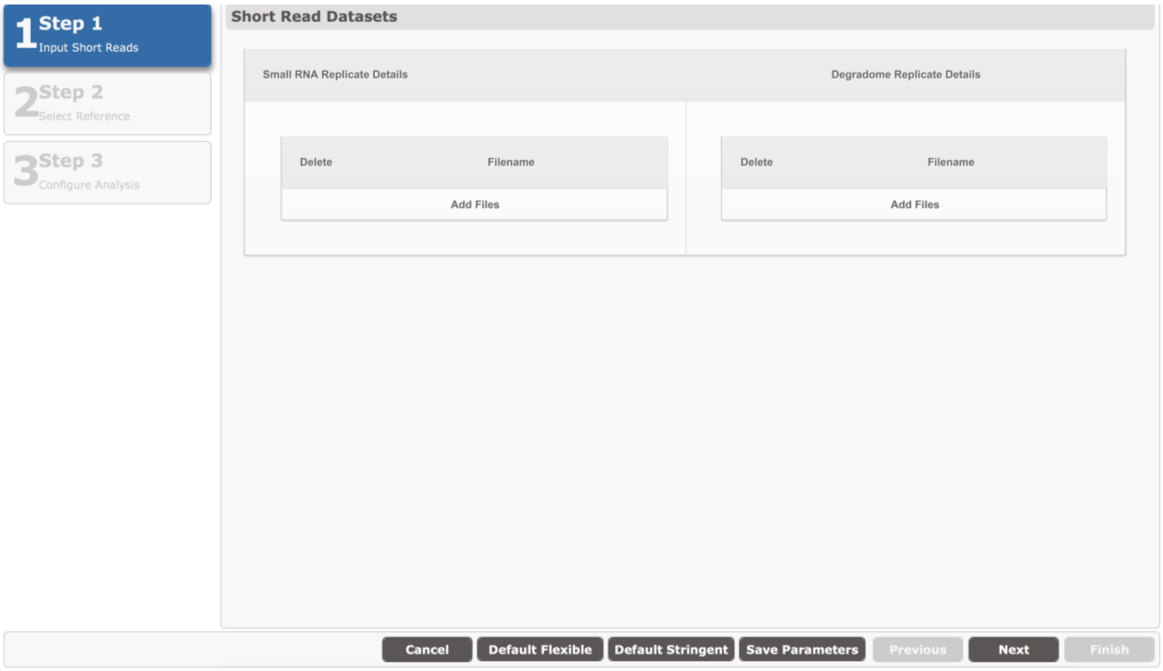

The first step is to add your short-read sequence files. Add one or more Small RNA and Degradome replicates by clicking on 'Add Files'.

One you have finished, click 'Next'.

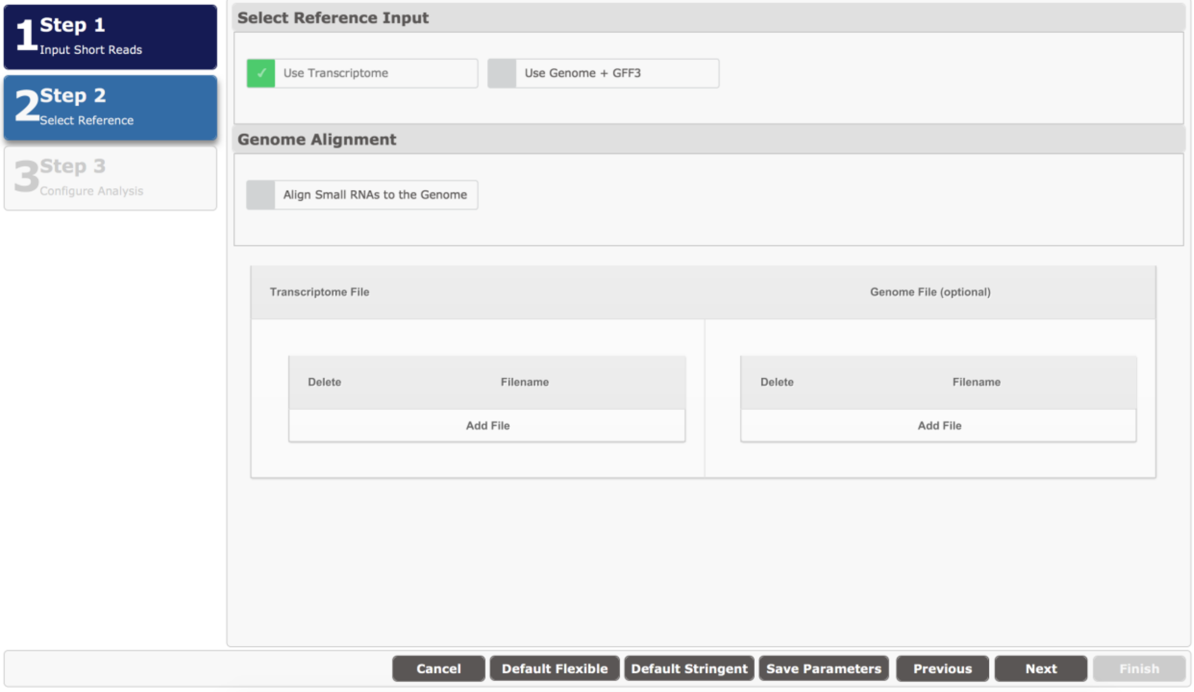

You now must input the reference sequence data. Select whether you want to use a transcriptome file or a GFF3 file and corresponding genome. Once you have made the selection you can choose to align the small RNA sequences to the genome (if provided).

Add the sequence data by using the 'Add File' button and then click 'Next' to continue.

Configuring analysis parameters

Once you have finished inputting the sequence data you will be provided with the following screen

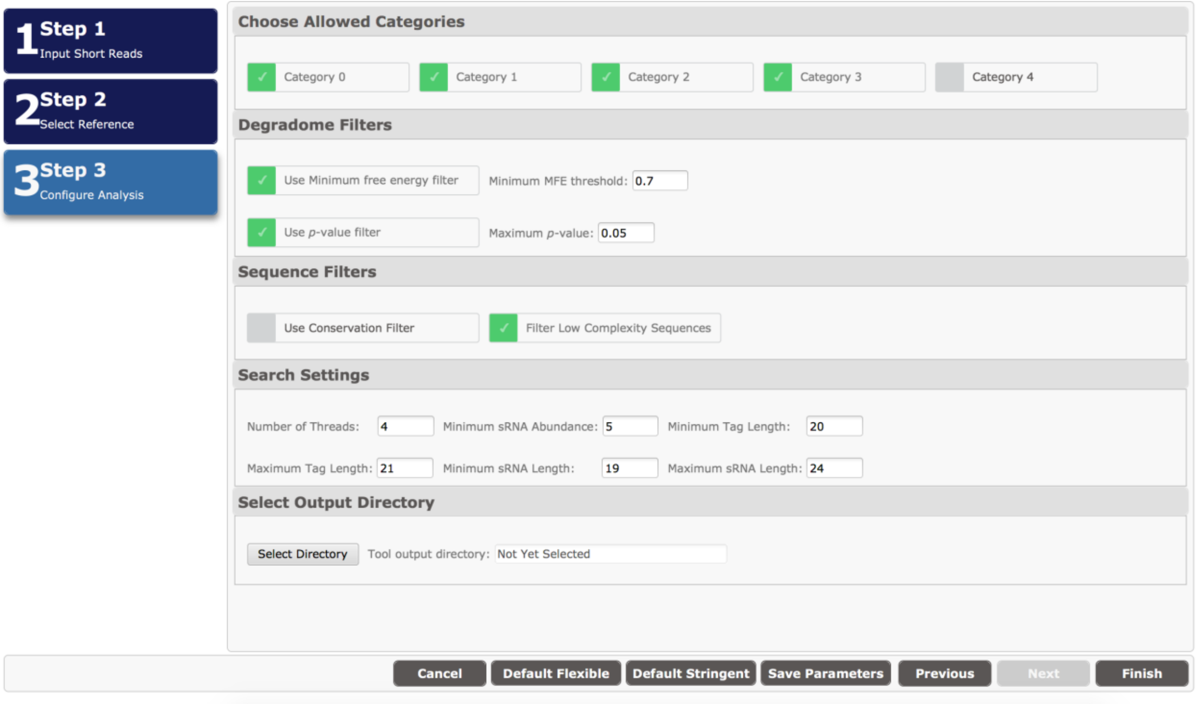

Here you can set the configuration parameters used during the analysis as discussed previously. You may click on the 'Default Flexible' or 'Default Stringent' buttons to load the default configurations. Optionally, you can save the configuration by clicking 'Save Parameters'.

Once you have finished, click on the 'Finish' button.

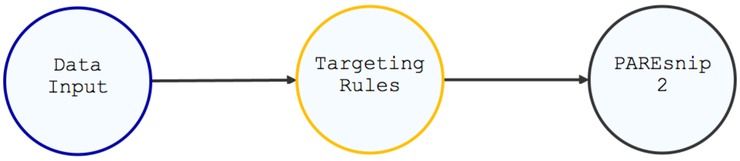

The ‘Data Input’ node will now turn blue indicating that it is ready.

Selecting targeting rules

Double click on the ‘Targeting Rules’ node and you will be given the following screen

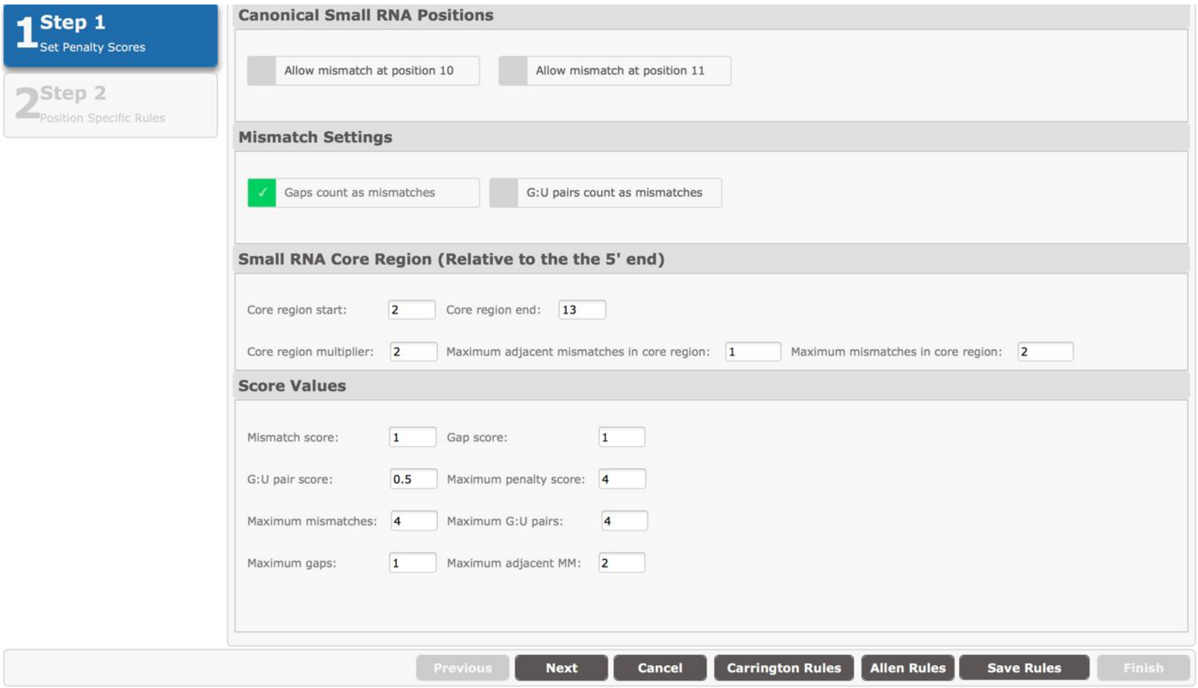

Here, you can set the chosen rules to be used during the analysis as described previously. Optionally, you may select to the use default Fahlgren and Carrington rules or the Allen et al. rules by clicking on the ‘Carrington Rules’ or ‘Allen Rules’ button, respectively. Optionally, you can save the selected targeting rules by clicking ‘Save Rules’.

Once you have finished, click ‘Next’, and you will be provided with the option to choose permissible and non-permissible mismatch positions.

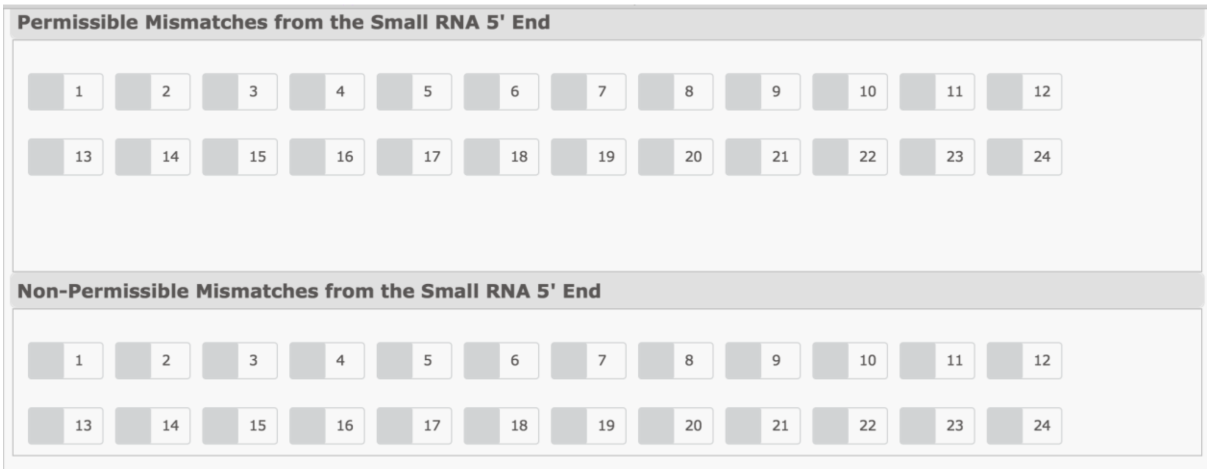

Just select the positions as appropriate or leave blank and click ‘Finish’.

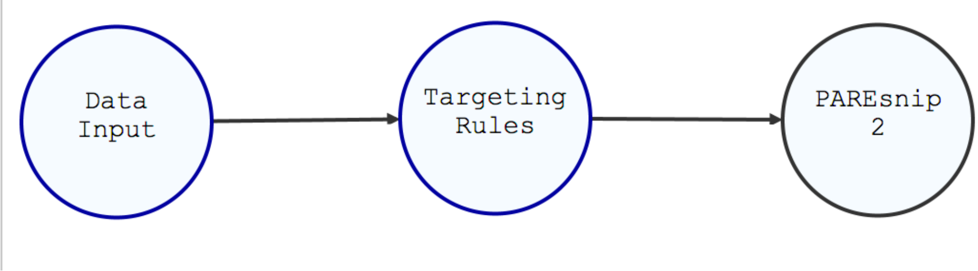

Both nodes will now be turned blue indicating that they are ready.

To start the analysis, click on ‘Open/Close Menu’ and then finally on ‘Begin Workflow’.

Launching PAREsnip2 from the command-line (CMD)

In order to execute the sRNA Workbench and PAREsnip2 from the command line, navigate to the directory that you extracted the sRNA Workbench files to. Then type:

```
java -jar Workbench.jar -tool paresnip2
```

If no options are entered, the usage instructions will be printed to the command line. An example of a complete instruction is given below:

```
Usage:
  java [-XmxNg] -jar /path/to/Workbench.jar -tool paresnip2 -parameters
/path/to/parameter/file -targeting_rules /path/to/targeting_rules/file
-srna_files /path/to/srna/file1 [/path/to/srna/file2] [...] -pare_files
/path/to/pare/file1 [/path/to/pare/file2] [...] -reference
/path/to/reference/file -output_dir /path/to/output/directory [-genome
/path/to/genome/file] [-gff3 /path/to/gff3/file]
Where:
 -XmxNg (optional but recommended) gives N GB of memory to the
Workbench process
```

Note: parameters in square brackets are optional. However, if using GFF and genome as reference then the –gff3 flag should provide the gff3 file and the – reference flag should provide the genome file.

Default parameter files that can be used or edited are found in the default parameters directory of the Workbench.

PAREsnip2 results output

Output column	Description
Record ID	Each prediction is given a numerical ID that ranges from 1 to N where N is the total number of predictions.
Short Read ID	The ID of the input small RNA sequence with the predicted target taken from the input file
Gene ID	The ID of the predicted target gene taken from the input file
Category	The category of the degradome peak from 0 to 4
Cleavage Position	The position on the transcript sequence that the small RNA is predicted to target. This will be the position that the degradome reads aligned
Fragment Abundance	The abundance of the degradome read that aligns to this position
Weighted Fragment Abundance	The abundance of the degradome read divided by the total number of transcripts that the sequence aligns
Normalized Fragment Abundance	Degradome reads per million normalized by library size
Short Read Abundance	Abundance of the small RNA in the input file
Normalized Short Read Abundance	Small RNA reads per million normalized by library size
Duplex	Visual representation of the alignment between the small RNA and messenger RNA
Alignment Score	The penalty score of the alignment based on the users chosen targeting criteria
Duplex MFE	The minimum free energy of the predicted duplex
Perfect MFE	The minimum free energy of a perfectly complemental duplex
MFE Ratio	precited duplex MFE / perfect duplex MFE
P-value	The interaction *P*-value reported by PAREsnip2
