# Supplementary figures and images for: PAREsnip2: a tool for high-throughput prediction of small RNA targets from degradome sequencing data using configurable targeting rules
Source: Nucleic Acids Res. 2018 Jul 11;46(17):8730–9. doi: 10.1093/nar/gky609 (PMC6158750; doi:10.1093/nar/gky609)

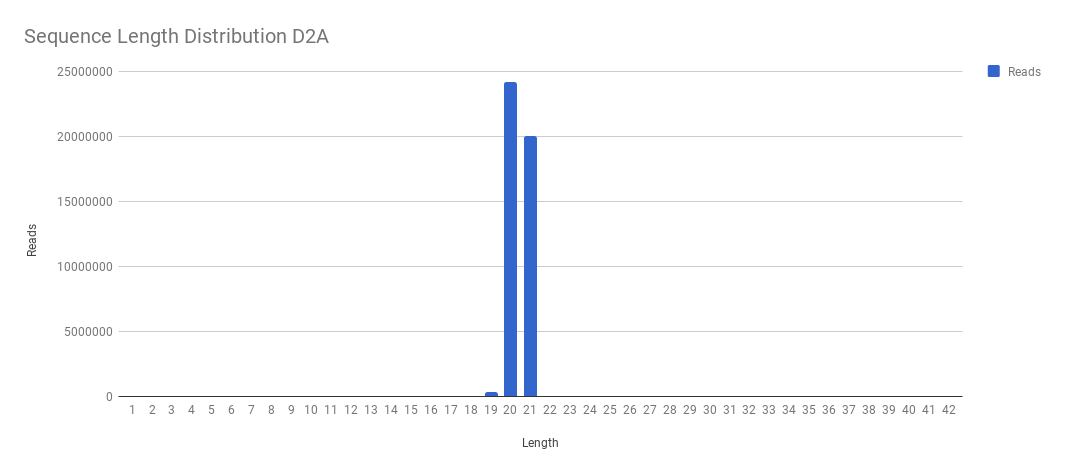

Supplement: Supplementary Data [file gky609_supplemental_files.zip › Supplementary_F1.png]

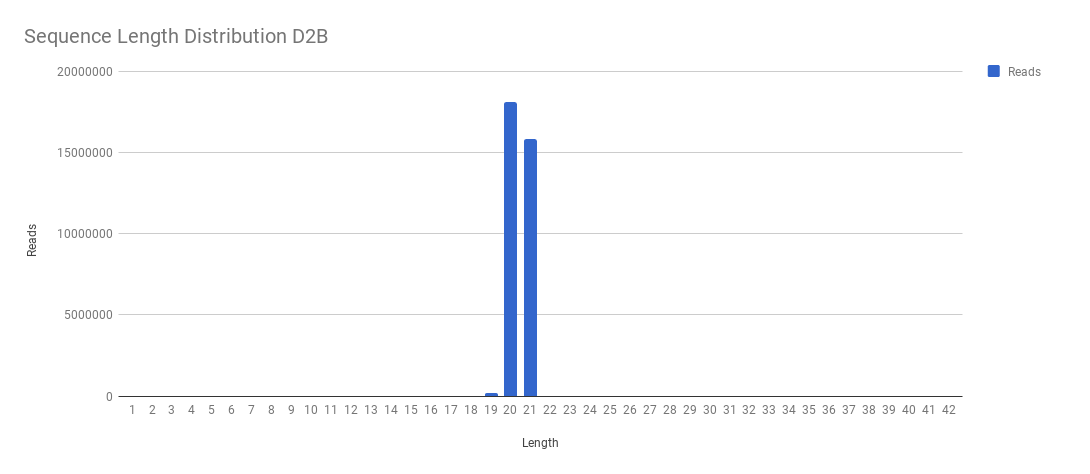

Supplement: Supplementary Data [file gky609_supplemental_files.zip › Supplementary_F2.png]

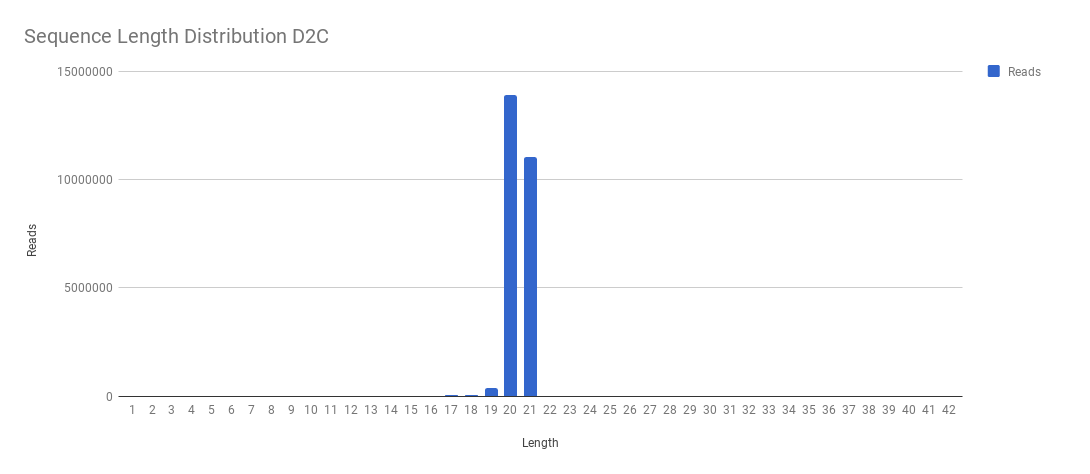

Supplement: Supplementary Data [file gky609_supplemental_files.zip › Supplementary_F3.png]
